# Supplementary material for: Content-rich biological network constructed by mining PubMed abstracts
Source: BMC Bioinformatics. 2004 Oct 8;5:147. doi: 10.1186/1471-2105-5-147 (PMC528731; doi:10.1186/1471-2105-5-147)
Supplement: Additional File 5 — The original Chilibot query results of the term "long-term potentiation (LTP)" and 22 other terms, limiting the latest references analyzed to the years 1990, 1995, 2000, and 2004. [file 1471-2105-5-147-S5.bz2 › chilibotAdditionalFile5/ltp2000/html/PKC_TAU.html]

 


 **PKC** and **TAU** 
  
Found 50 abstracts in PubMed,  **30 abstracts were retrieved and analyzed**.  


---

 Search Google  |
 PDF files only 
|  EDU domain only 

---

**Interactive relationship** (e.g. stimulation, inhibition, etc)

- Glycogen synthase kinase 3 GSK 3beta phosphorylates  **tau**  and is inhibited via protein kinase C  **PKC** .  Ref: 11129110 J Neural Transm, 2000
- By contrast, treatment with either TPA or the  **PKC**  inhibitor staurosporine did not induce  **tau**  phosphorylation and induced neurite elaboration.  Ref: 9568534 Int J Dev Neurosci, 1997
- Membrane targeted 14 3 3  **tau**  was more effective than wild type 14 3 3  **tau**  in suppressing  **PKC**  theta dependent IL 2 promoter activity, suggesting that 14 3 3  **tau**  inhibits the function of  **PKC**  theta not only by preventing its translocation to the membrane but also by associating with it.  Ref: 8816492 Mol Cell Biol, 1996
- By contrast, downregulation of either  **PKC**  epsilon or  **PKC**  alpha reduced immunoreactivity towards the phosphate independent anti  **tau**  antibodies 5E2 and JM, suggesting that both of these isoforms participated in regulation of  **tau**  steady state levels.  Ref: 9253654 Int J Dev Neurosci, 1997
- Downregulation of  **PKC**  epsilon by both of these methods reduced PHF I and ALZ 50 immunoreactivity, suggesting that this  **PKC**  isoform, perhaps via downstream kinase cascades, regulated  **tau**  phosphorylation events that normally generate these epitopes.  Ref: 9253654 Int J Dev Neurosci, 1997
- Transient overexpression of 14 3 3  **tau**  suppressed stimulation of the interleukin 2 IL 2 promoter mediated by cotransfected wild type or constitutively active  **PKC**  theta, as well as by endogenous  **PKC**  in ionomycin and or phorbol ester stimulated cells.  Ref: 8816492 Mol Cell Biol, 1996
- These epitopes were masked by higher  **PKC**  concentrations, suggesting the presence of multiple  **tau**  phosphorylation sites for which  **PKC**  exhibited differing affinities and or conformational alterations in  **tau**  induced by sequential  **PKC**  mediated phosphorylation.  Ref: 10081606 Cell Mol Neurobiol, 1999
- Overexpression of wild type 14 3 3  **tau**  also inhibited phorbol ester induced  **PKC**  theta translocation from the cytosol to the membrane in Jurkat cells, while a membrane targeted form of 14 3 3  **tau**  caused increased localization of  **PKC**  theta in the particulate fraction in unstimulated cells.  Ref: 8816492 Mol Cell Biol, 1996
- Hyperactivation of protein kinase C  **PKC**  in intact neuroblastoma cells by several methods increases site specific  **tau**  phosphorylation as shown by increases in paired helical filament I PHF I and ALZ 50 but not AT 8 immunoreactivity.  Ref: 9253654 Int J Dev Neurosci, 1997
- Elevated expression of  **PKC**  alpha, gamma, zeta, epsilon, lambda, and  **tau**  were observed in TCDD transformed cells, indicating a possible association of altered expression of  **PKC**  isozymes with TCDD induced transformation of human cells.  Ref: 9734276 Chemosphere, 1998
- Site specific phospho  **tau**  immunoreactivity was increased in additive and synergistic manners by treatment of injected cells with TPA which activates  **PKC** , calcium ionophore which activates calcium dependent kinases, and wortmannin which inhibits PIP3 kinase.  Ref: 10081608 Cell Mol Neurobiol, 1999
- Direct interaction between protein kinase C theta  **PKC**  theta and 14 3 3  **tau**  in T cells 14 3 3 overexpression results in inhibition of  **PKC**  theta translocation and function.  Ref: 8816492 Mol Cell Biol, 1996
- **PKC**  theta and 14 3 3  **tau**  coimmunoprecipitated from Jurkat T cells, and recombinant 14 3 3  **tau**  interacted directly with purified  **PKC**  theta in vitro.  Ref: 8816492 Mol Cell Biol, 1996
- These results suggested that the modulation of MAP 2 and  **Tau**  protein may reflect changes of  **PKC**  and CaM kinase,.  Ref: 9199666 J Histochem Cytochem, 1997

**Parallel relationship** (e.g. studied together, co-existance, homology, etc.)

- Developmental regulation and  **PKC**  dependence of Alzheimer s type  **tau**  phosphorylations in cultured fetal rat hippocampal neurons.  Ref: 9602100 Brain Res Dev Brain Res, 1998
- The cardiac expression of a range of  **PKC**  isoforms  **PKC**  alpha,  **PKC**  omega,  **PKC**  epsilon,  **PKC**  gamma, and  **PKC**   **tau**  was examined by immuno blotting.  Ref: 9713323 J Endocrinol, 1998
- Adult female Wistar rats were treated with estrogen and bromocriptine and their pituitary glands were removed for analysis of the expression of tubulin, MAP 2,  **Tau**  protein, protein kinase C  **PKC** , and calcium calmodulin CaM kinase.  Ref: 9199666 J Histochem Cytochem, 1997
- Western blot analysis showed that estrogen increased and bromocriptine decreased the expression of  **PKC**  alpha, beta 1, beta 2, CaM kinase alpha, beta, MAP 2, and  **Tau**  protein.  Ref: 9199666 J Histochem Cytochem, 1997
- Our previous data showed that at least five  **PKC**  isoforms alpha, delta, zeta, lambda and  **tau**  were present in the decidualization.  Ref: 9551254 Chin J Physiol, 1997
- We analyzed the effects of PKNalpha and protein kinase C  **PKC**  on phosphorylation of  **tau**  protein by glycogen synthase kinase GSK 3beta using monoclonal antibodies AT8, AT180, and AT270.  Ref: 10873588 Biochem Biophys Res Commun, 2000
- Here, we examined potential physical and functional interactions between  **PKC**  theta, a calcium independent  **PKC**  enzyme which is expressed selectively in T lymphocytes, and the 14 3 3  **tau**  isoform in vitro and in intact T cells.  Ref: 8816492 Mol Cell Biol, 1996
- Selective activation by bryostatin 1 demonstrates unique roles for  **PKC**  epsilon in neurite extension and  **tau**  phosphorylation.  Ref: 9568534 Int J Dev Neurosci, 1997
- As certain muscarinic receptors are linked to  **PKC** , we examined the effect of a range of agonists on GSK 3beta phosphorylation of  **tau** .  Ref: 11129110 J Neural Transm, 2000
- We identified among these proteins an 8 0 kDa and the  **tau**  protein as specific substrates for protein kinase A PKA, MARCKS, and neurogranin as specific substrates for protein kinase C  **PKC** , and prostaglandin D synthase as substrate for casein kinase II CKII.  Ref: 10226777 Hippocampus, 1999
- The individual and sequential influence of protein kinase C  **PKC** , protein kinase A PKA and mitogen activated protein kinase MAP kinase on human brain  **tau**  was examined.  Ref: 10081606 Cell Mol Neurobiol, 1999
- These findings provide additional evidence for a unique role of  **PKC**  epsilon in the regulation of  **tau**  phosphorylation and neuronal differentiation, and demonstrate that bryostatin 1 can function under certain conditions as a selective  **PKC**  epsilon activator even following long term treatment.  Ref: 9568534 Int J Dev Neurosci, 1997
- These findings indicate that  **PKC**  phosphorylates  **tau**  via the MAP kinase pathway, but that PKM can bypass this requirement, therefore demonstrating that distinct intracellular pathways can be mediated by  **PKC**  and PKM.  Ref: 10629766 Brain Res, 1999
- The above roles were apparently unique for  **PKC**  epsilon and  **PKC**  alpha, since activation of multiple  **PKC**  isoforms by phorbol ester treatment and or other calcium dependent kinase s by ionophore mediated calcium influx could not compensate for downregulation of  **PKC**  alpha or  **PKC**  epsilon in maintaining  **tau**  steady state levels or PHF I ALZ 50 immunoreactivity, respectively.  Ref: 9253654 Int J Dev Neurosci, 1997
- This did not represent a general inhibition of activation events, since  **PKC**  independent but calcium dependent activation of an IL 4 promoter element was not inhibited by 14 3 3  **tau**  under similar conditions.  Ref: 8816492 Mol Cell Biol, 1996
- It is suggested that fragmentation of  **PKC**  delta during the process of apoptosis results in the phosphorylation and inactivation of GSK 3 and consequently inhibition of the phosphorylation of  **tau** .  Ref: 10708767 FEBS Lett, 2000
- Immunoblot analysis demonstrated that both conventional  **PKC**  alpha, beta, gamma and atypical  **PKC**  isozymes zeta,  **tau**  were present in HEK cell membranes.  Ref: 9346296 Eur J Biochem, 1997
- We examined the downstream consequences of  **PKC**  activation by the phorbol ester TPA and by ionophore A23187 mediated calcium influx on phosphorylation of the microtubule associated protein  **tau** .  Ref: 10629766 Brain Res, 1999
- Our objective was to test whether bIFN  **tau**  could block PGF 2 alpha production and synthesis of phospholipase A 2 PLA 2 and cyclooxygenase 2 COX 2 enzymes induced by a protein kinase C  **PKC**  stimulator phorbol 1 3 dibutyrate.  Ref: 10906045 Biol Reprod, 2000
- In the present study, the influence of  **PKC**  on  **tau**  metabolism was further examined by isoform specific antisense oligonucleotide mediated  **PKC**  downregulation in human SH SY 5Y neuroblastoma cells and by generation of stably transfected subclones expressing isoform specific anti  **PKC**  mRNA sequences.  Ref: 9253654 Int J Dev Neurosci, 1997
- Since previous studies have implicated this  **PKC**  isoform in phosphorylation of the microtubule associated protein  **tau**  and in neuritogenesis, we examined the consequences of long term bryostatin treatment on these phenomena.  Ref: 9568534 Int J Dev Neurosci, 1997
- Prior phosphorylation by  **PKC**  enhanced the nature and extent of AD like  **tau**  antigenicity generated by subsequent incubation with MAP kinase yet inhibited that generated by subsequent incubation with PKA.  Ref: 10081606 Cell Mol Neurobiol, 1999
- IFN  **tau**  may regulate the PGF 2a synthetic pathway by reducing activity of  **PKC**  or  **PKC**  mediated events.  Ref: 10764976 Domest Anim Endocrinol, 2000
